# Efficacy of β–Glucan From Microalgae on the Intestinal Health and Growth of Nursery Pigs

**DOI:** 10.1111/asj.70056

**Published:** 2025-04-21

**Authors:** Naiana E. Manzke, Alexa R. Gormley, Young Ihn Kim, Wanpuech Parnsen, Sung Woo Kim

**Affiliations:** ^1^ Department of Animal Science North Carolina State University Raleigh North Carolina USA

**Keywords:** antibiotics, intestinal health, microalgae, nursery pigs, β–glucan

## Abstract

This study aimed to investigate the efficacy of β‐glucan from microalgae (Algamune, Algal Scientific, Plymouth, MI, USA) on the intestinal health and growth of nursery pigs. One hundred sixty nursery pigs (6.0 ± 1.6 kg BW) were assigned to four treatments arranged in a 2 × 2 factor: antibiotic use and β‐glucan supplementation (0.02% Algamune). Diets were fed for 5 weeks, at which eight pigs from each treatment were selected for tissue sample collection. During the overall experimental period, antibiotics improved (*p* < 0.05) ADG and G:F. In Week 5, β‐glucan supplementation increased (*p* < 0.05) the ADG of pigs without antibiotics, whereas β‐glucan supplementation had no effect in pigs with antibiotics. In Phase 3, β‐glucan supplementation tended to increase (*p* = 0.061) ADFI of pigs without antibiotics, whereas β‐glucan supplementation had no effect in pigs with antibiotics. Both antibiotics and β–glucan increased (*p* < 0.05) villus height in the duodenum. Supplementation of β–glucan reduced (*p* < 0.05) IgA in the jejunum and tended to reduce (*p* = 0.053) IgA in the ileum. In conclusion, β–glucan from microalgae may encourage growth and feed intake of nursery pigs by improving intestinal health when antibiotics are not used in the diets.

## 1 Introduction

In the past, antibiotics were commonly used as growth promoters in pig production to minimize the negative effects associated with the weaning process. However, concerns of widespread antimicrobial resistance have reduced and banned the use of antibiotics for growth promotion globally (Prestinaci, Pezzotti, and Pantosti 2015). This reduction in the use of antibiotics as growth promoters has led pig producers to look for new alternatives to minimize the compromised growth performance due to weaning stress, when antibiotics are not used. Some such alternatives include acidifiers (Ahmed et al. 2014; Upadhaya et al. 2018), direct fed microbials (Davis et al. 2007; Lewton et al. 2022), and prebiotics, probiotics, and postbiotics (Duarte and Kim 2024; San Andres et al. 2019; Xiang et al. 2020).

β‐Glucan is a polymer of glucose linked by various types of β‐glycosidic bonds and is the major structural component of the cell wall of yeast, fungi, some bacteria, plants, and microalgae (Du et al. 2019). The specific β‐glucan structure is dependent on the source, and the variation in structure can affect solubility and biological activity. Microalgae ( *Euglena gracilis* ) can accumulate high quantities of β‐1,3‐glucan intracellularly, which may compose over 90% dry weight of the cell (Barsanti and Gualtieri 2020). Previous studies indicate that the inclusion of β‐glucans from various sources in pig feeds may provide prebiotic and immunomodulatory effects (K. Kim, Ehrlich, et al. 2019; Reilly et al. 2010; Sweeney et al. 2012). Specifically, β‐glucan has been shown to stimulate the immune response in both in vivo and in vitro conditions, as evidenced by an increase in pro‐inflammatory cytokine release in response to macrophage activation as a defense against potential infection (Li et al. 2006; Volman, Ramakers, and Plat 2008; Young et al. 2001). Dietary β‐glucan has also been found to improve epithelial barrier integrity and reduce inflammation under challenge conditions, in mice (Li et al. 2022; Zhang et al. 2023).

Aside from β‐glucan, microalgae also contain cellulose, fucan, xylan, and mannan within their cell walls, that also can have bioactive properties (Baudelet et al. 2017; Cheng and Kim 2022). Extensive evidence supports the prebiotic effects of microalgae, as the polysaccharides within the cell walls can be digested by the intestinal microbiota, serving as an energy source for microbes and the host through fermentation to produce short‐chain fatty acids (SCFA) (Raposo, De Morais, and Bernardo de Morais 2013). The presence of SCFA can greatly benefit the host by modulating energy metabolism (Zhou et al. 2021), serving as an energy source for colonic cells (Kien et al. 2007), encouraging an increase in potentially beneficial microbial species (Lingbeek et al. 2021), maintaining intestinal epithelial integrity (Feng et al. 2018; Peng et al. 2009), and inhibiting intestinal inflammation through regulation of the immune system and cytokine release (Park et al. 2015). Considering the potential positive effects of β‐glucans on immune system activation and modulation of the inflammatory state under stress conditions and the functional roles of other bioactive compounds found within the cell walls, the use of dietary microalgae could be effective in mitigating the negative effects associated with weaning on the intestine of nursery pigs.

Therefore, the hypothesis of this study was that β‐glucan from microalgae may stimulate an intestinal immune response, possibly through improved intestinal health, consequently enhancing growth performance of nursery pigs. The objective of this study was to determine the effect of β‐glucan extracted from microalgae on the intestinal immune response, intestinal health parameters, and growth performance of nursery pigs fed diets with or without antibiotics.

## 2 Materials and Methods

The animal experimental protocol used in this study was approved by the North Carolina University Animal Care and Use Committee.

### 2.1 Source of β‐Glucan

The β‐glucan used in this study is commercially available as Algamune (Algal Scientific Corp., Plymouth, MI, USA), which was obtained from the extraction of microalgae *Euglena gracilis* . Algamune contained 55% β‐1,3‐glucan, 25% crude protein, 4% crude fat, 7% ash, and 7% moisture.

### 2.2 Animals and Experimental Diets

One hundred sixty newly weaned crossbred barrows and gilts (Smithfield Premium Genetics, Rose Hill, NC, USA) with initial body weight (BW) of 6.0 ± 1.6 kg were housed in 40 pens with four pigs per pen, with all pigs being of the same sex at the Swine Evaluation Station (Clayton, NC, USA) of North Carolina State University. There were 10 pens (five barrow and five gilt pens) per treatment. Pigs were fed the experimental diets for 5 weeks, divided into three phases: Phase 1 (Week 1), Phase 2 (Weeks 2 and 3), and Phase 3 (Weeks 4 and 5).

Pigs were randomly allotted to their four respective treatments using a randomized complete block design with initial BW and sex as blocks. Treatments were arranged in 2 × 2 factors (Table 1) with antibiotics (no antibiotics or a combination of antibiotics) and β‐glucan (0.00% or 0.02% of Algamune; 0.00% or 0.01% of β‐glucan, respectively) as the factors. Antibiotics utilized were the combined use of Pennchlor 100 (2000 mg/kg) and Denagard (1750 mg/kg) in Phase 1 and Mecadox 10 (1250 mg/kg) in Phases 2 and 3. All diets were formulated following the procedures described in Kim and Hansen (2023) to contain nutrients meeting the or exceeding requirements suggested by the NRC (2012).

**TABLE 1:** Composition of experimental diets (as‐fed basis).

	Phase 1	Phase 2	Phase 3
Feedstuff, %			
Corn	32.14	44.94	62.21
Soybean meal (48% CP)	25.00	29.00	32.00
Whey permeate	25.00	15.00	0.00
Poultry meal	6.00	2.00	0.00
Blood plasma	6.00	3.00	0.00
L‐Lys HCl	0.37	0.35	0.35
DL‐Met	0.20	0.15	0.11
L‐Thr	0.10	0.09	0.09
Sodium chloride	0.22	0.22	0.22
Dical P (22% Ca, 18% P)	0.30	0.75	1.10
Limestone	0.85	0.93	0.85
Vitamin premix^a^	0.03	0.03	0.03
Trace mineral premix^b^	0.15	0.15	0.15
Supplement^c^	0.65	0.40	0.40
Poultry fat	3.00	3.00	2.50
Calculated composition			
DM, %	90.78	90.14	89.36
ME, kcal/kg	3461	3433	3407
CP, %	24.25	21.99	20.83
SID^d^ Lys, %	1.50	1.35	1.23
SID Met + Cys, %	0.82	0.74	0.68
SID Trp, %	0.27	0.25	0.22
SID Thr, %	0.88	0.79	0.73
Ca, %	0.85	0.80	0.70
STTD^e^ P, %	0.45	0.40	0.33
Total P, %	0.66	0.63	0.60

^a^ The vitamin premix provided the following per kilogram of complete diet: 6613.8 IU of vitamin A as vitamin A acetate, 992.0 IU of vitamin D_3_, 19.8 IU of vitamin E, 2.64 mg of vitamin K as menadione sodium bisulfate, 0.03 mg of vitamin B_12_, 4.63 mg of riboflavin, 18.52 mg of D‐pantothenic acid as calcium pantothenate, 24.96 mg of niacin, and 0.07 mg of biotin.

^b^ The trace mineral premix provided the following per kilogram of complete diet: 4.0 mg of Mn as manganous oxide, 165 mg of Fe as ferrous sulfate, 165 mg of Zn as zinc sulfate, 16.5 mg of Cu as copper sulfate, 0.30 mg of I as ethylenediamine dihydroiodide, and 0.30 mg of Se as sodium selenite.

^c^ Supplement = phase 1: β‐glucan (0.02% of Algamune, Algal Scientific Corp., Plymouth, MI)); antibiotics (0.20% of Pennchlor 100; 0.18% of Denagard; 0.25% of zinc oxide), phase 2: β‐glucan (0.02% of Algamune); antibiotics (0.13% of Mecadox 10; 0.25% of zinc oxide), phase 3: β‐glucan (0.02% of Algamune); antibiotics (0.13% of Mecadox 10; 0.25% of zinc oxide).

^d^ SID, standardized ileal digestible.

^e^ STTD P, standardized total tract digestible phosphorus.

Pens had solid concrete flooring and were equipped with one self‐feeder and a nipple to allow ad libitum access to feed and water throughout the entire experimental period. BW and feed intake were measured weekly for evaluation of growth performance including average daily gain (ADG), average daily feed intake (ADFI), and gain:feed ratio (G:F).

### 2.3 Blood Sampling

On d 35, blood was collected from the pig with the median initial BW per pen. Blood samples (7 mL) were collected aseptically from the jugular vein into vacutainers tubes (BD, Franklin Lakes, NJ, USA) containing EDTA at 10:00 h. Plasma was separated by centrifugation at 1500 × *g* at 4°C for 10 min, and the samples were subsequently frozen at −80°C for further analysis.

Plasma samples were used to measure immunological responses including immunoglobulins A and G (IgA and IgG) and tumor necrosis factor‐α (TNF‐α), as indicators of overall immune status. The total concentration of immunoglobulins were measured using an ELISA kit (number E100‐102; Bethyl Laboratories, Montgomery, TX, USA) as previously described by Chaytor et al. (2011). Plasma samples were diluted 1:40,000 or 1:1000 for determination of IgA and IgG, respectively. Absorbance was read at 450 nm using a Synergy HT plate reader (BioTek Instruments, Inc., Winooski, VT, USA) and Gen5 data analysis software (BioTek Instruments, Inc.). Samples were quantified relative to a standard curve based on known amounts of porcine IgA and IgG. The detection limits for the ELISA were 15.6 to 1000 ng/mL and 7.8 to 500 ng/mL, for IgA and IgG, respectively.

Plasma TNF‐α concentration was measured using an ELISA kit (PTA00; R&D Systems, Minneapolis, MN, USA) as previously described by Shen et al. (2009). A total of 50 μL of standard, control, or sample were added to microplate wells coated with a monoclonal antibody for porcine TNF‐α. Absorbance was read at 450 and 540 nm, and the detection limit range for the TNF‐α ELISA was 2.8 to 5.0 pg/mL.

### 2.4 Intestinal Sampling

At the end of phase 3, 32 pigs (four barrow pens and four gilt pens per treatment, *n* = 8) of the median BW were selected and humanely euthanized. The gastrointestinal tract was promptly identified and removed. The jejunum was gently squeezed to remove the digesta and was immediately placed on ice for further measurement of viscosity. Middle portions of the duodenum (10 cm after the pyloric valve) and jejunum (3 m prior to the ileocecal junction) of each pig were collected, and 10‐cm sections were obtained and divided to two subsections of 5 cm. The first 5 cm of each section was gently flushed with sterile saline and submerged in a 10% formaldehyde–phosphate buffer in an individual container for further microscopic evaluation of intestinal morphology. The remaining 5 cm of each section distal portion of the duodenum and jejunum, as well as 5 cm of the ileum, was also obtained to collect 1.5 mL of mucosal samples, which were immediately placed in liquid nitrogen until the conclusion of sampling when they were subsequently transferred to −80°C freezer for further analysis.

### 2.5 Intestinal Morphology

The formalin‐fixed jejunum and colon sections were trimmed and placed in cassettes. The North Carolina State University Histopathology Laboratory (College of Veterinary Medicine, Raleigh, NC, USA) embedded the samples in paraffin, thin‐sectioned, and secured them on microscope slides with hematoxylin and eosin staining. The slides were evaluated under a Sony CCD video camera attached to a microscope under 40× magnification. An average of 10 villus height, villus width, and crypt depth were measured in each slide following the methodology described by Baker et al. (2024).

### 2.6 Viscosity of Digesta

After the samples were collected, jejunal digesta was immediately taken to the laboratory to measure the viscosity based on the methods described by Jang et al. (2024). Each sample was replicated four times. The digesta were homogenized and transferred to 15‐mL tubes. The tubes were centrifuged at 1500 × *g* for 5 min at 4°C, and then, the supernatant was again centrifuged at 10,000 × *g* for 10 min at 4 °C. The second supernatant was placed in ice to measure viscosity immediately. A viscometer (DV2T, Brookfield Engineering Laboratories, Inc., MA, USA) was set at 25°C, and 1.0 mL of the supernatant was placed in the viscometer.

### 2.7 Immune Parameters and Oxidative Stress

Each mucosa sample (0.50 g) was homogenized with 1 mL of PBS solution and centrifuged for 10 min at 15,000 × *g*. A total of 2 mL of supernatant was obtained for total protein calculation by BCA assay (Peace et al. 2011). The total concentration of immunoglobulins and TNF‐α were measured as previously mentioned in the text.

Mucosa samples were diluted by a factor of 500, 50, and 50 for duodenum, jejunum, and ileum, respectively, for determination of IgA, and 1000 for duodenum, jejunum, and ileum, for determination of IgG.

Concentrations of protein carbonyl were measured via the protein carbonyl ELISA kit (Cell Biolabs, San Diego, CA, USA) as previously described by Deng, Duarte, and Kim (2023). The protein carbonyl present in the sample or standard were derivatized to dinitrophenyl (DNP) hydrazine and probed with an anti‐DNP antibody, then incubated with a secondary antibody. Finally, substrate and stop solutions were added. Samples and standards were read at 450 nm, and protein carbonyl concentrations were calculated by comparing sample absorbance reads with the protein carbonyl standard curve that was determined using the standard absorbance reads. The detection limit for protein carbonyl was 0.375 nmol/mg.

### 2.8 Statistical Analysis

Data were analyzed using the MIXED procedure of SAS (SAS Inc., Cary, NC, USA) utilizing a randomized complete block design. The pen was considered as the experimental unit. The effects of antibiotics, β‐glucan supplementation, and the interaction between antibiotics and β‐glucan supplementation were considered the fixed effects, whereas the initial BW and sex blocks were considered the random effects. When an interaction between the use of antibiotics and β‐glucan supplementation was significant or tended to be significant, a pairwise comparison was made using the PDIFF option in SAS. Probability values less than 0.05 were considered statistically significant and between 0.05 and 0.10 were considered to be tendency.

## 3 Results

3.1 Growth Performance

Initial BW did not differ among treatments (Table 2). During the overall experimental period, the use of antibiotics increased (*p* < 0.05) ADG, ADFI, and G:F. There was no effect of β‐glucan on growth performance seen in the overall experimental period. In Week 3, the supplementation of β‐glucan tended to increase (*p* = 0.052) ADG of pigs in diets without antibiotics, whereas the ADG of pigs was not affected by the combined supplementation of β‐glucan and antibiotics. In Week 5, the supplementation of β‐glucan increased (*p* < 0.05) ADG of pigs in diets without antibiotics, whereas the ADG of pigs was not affected by the combined supplementation of β‐glucan and antibiotics. In the overall experimental period, supplementation of β‐glucan tended to increase (*p* = 0.093) ADG of pigs in diets without antibiotics, whereas the ADG of pigs was not affected by the combined supplementation of β‐glucan and antibiotics. In Phase 3, supplementation of β‐glucan tended to increase (*p* = 0.061) ADFI of pigs in diets without antibiotics, whereas the ADFI of pigs was not affected by the combined supplementation of β‐glucan and antibiotics. In Week 3 and Phase 2, supplementation of β‐glucan tended to increase (*p* = 0.075 and 0.055, respectively) G:F of pigs in diets without antibiotics, whereas the G:F of pigs was not affected by the combined supplementation of β‐glucan and antibiotics.

**TABLE 2:** Growth performance of nursery pigs fed diets supplemented with antibiotics and β‐glucan from microalgae.

Antibiotics^a^	No		Yes		SEM	*p*		
β‐glucan^b^	No	Yes	No	Yes		Antibiotic	β‐glucan	Interaction
BW, kg								
Initial	6.0	6.0	6.1	6.0	0.5	0.826	0.976	0.735
Week 1	6.4	6.5	6.8	6.9	0.5	0.026	0.508	0.953
Week 2	7.8	8.0	8.7	8.7	0.6	0.002	0.612	0.646
Week 3	10.1	10.9	11.6	11.3	0.8	0.015	0.525	0.191
Week 4	13.2	14.3	15.8	15.5	1.1	0.001	0.521	0.171
Week 5	16.4	18.1	20.1	19.6	1.3	0.001	0.377	0.104
ADG, g/day								
Phase 1 (Week 1)	54	64	98	119	15	0.002	0.305	0.719
Week 2	199	219	281	267	26	0.001	0.889	0.340
Week 3	330^B^	405^A^	410^A^	371^AB^	36	0.431	0.538	0.052
Phase 2 (Weeks 2 and 3)	265^B^	312^AB^	346^A^	319^A^	28	0.035	0.617	0.071
Week 4	448	487	606	590	49	< 0.001	0.729	0.404
Wek 5	454^b^	549^a^	608^a^	589^a^	33	0.002	0.186	0.049
Phase 3 (Weeks 4 and 5)	451	518	607	589	35	< 0.001	0.347	0.110
Overall	297^C^	345^B^	401^A^	387^A^	26	< 0.001	0.347	0.093
ADFI, g/day								
Phase 1 (Week 1)	124	120	128	140	12	0.302	0.711	0.471
Week 2	282	296	345	340	24	0.002	0.750	0.548
Week 3	484	522	570	539	40	0.048	0.883	0.179
Phase 2 (Weeks 2 and 3)	383	409	457	440	32	0.010	0.823	0.260
Week 4	665^c^	752^bc^	885^a^	829^ab^	59	< 0.001	0.651	0.047
Week 5	811	928	1033	1011	73	0.001	0.272	0.110
Phase 3 (Weeks 4 and 5)	738^C^	840^B^	959^A^	920^AB^	64	< 0.001	0.391	0.061
Overall	473	524	592	572	38	0.001	0.489	0.115
G:F								
Phase 1 (Week 1)	0.39	0.46	0.74	0.85	0.10	< 0.001	0.363	0.836
Week 2	0.68	0.74	0.82	0.78	0.04	0.043	0.819	0.261
Week 3	0.66^B^	0.78^A^	0.71^AB^	0.70^AB^	0.05	0.684	0.157	0.075
Phase 2 (Weeks 2 and 3)	0.67^B^	0.76^A^	0.75^A^	0.73^AB^	0.04	0.446	0.249	0.055
Week 4	0.69	0.65	0.68	0.71	0.04	0.455	0.854	0.372
Week 5	0.57	0.59	0.59	0.59	0.02	0.435	0.497	0.471
Phase 3 (Weeks 4 and 5)	0.62	0.62	0.63	0.64	0.02	0.285	0.888	0.687
Overall	0.62	0.66	0.68	0.68	0.01	0.017	0.222	0.259

^a^ Antibiotics, phase 1: 0.20% of Pennchlor 100, 0.18% of Denagard, 0.25% of zinc oxide; and phase 2 and 3: 0.13% of Mecadox 10; 0.25% of zinc oxide.

^b^ β‐glucan, 0.02% of Algamune (Algal Scientific Corp., Plymouth, MI).

^a–c^ Within a row, means lacking common superscripts are different at *p* < 0.05.

^A,B^ Within a row, means lacking common superscripts are different at 0.05 ≤ *p <* 0.10.

Pigs fed antibiotics and β‐glucan had an increased (*p* < 0.05) villus height (214 to 243 and 214 to 262 μm, respectively) in the duodenum (Table 3). In addition, antibiotics tended to increase (*p* = 0.077) crypt depth in the duodenum. The treatments did not affect morphology of the villi and crypts in the jejunum. Viscosity of jejunal digesta in the jejunum was not affected by treatments.

**TABLE 3:** Intestinal morphology and jejunal digesta viscosity of nursery pigs fed diets supplemented with antibiotics and β‐glucan from microalgae.

Antibiotics^a^	No		Yes		SEM	*p*		
β‐glucan^b^	No	Yes	No	Yes		Antibiotic	β‐glucan	Interaction
Duodenum								
Villus height, μm	214	262	243	306	17	0.040	0.003	0.667
Villus width, μm	81	79	81	92	4	0.133	0.306	0.138
Crypt depth, μm	131	143	151	171	13	0.077	0.231	0.764
VH:CD	1.69	1.84	1.67	1.94	0.15	0.835	0.229	0.734
Jejunum								
Villus height, μm	233	238	223	209	14	0.181	0.755	0.507
Villus width, μm	88	90	87	90	5	0.587	0.805	0.854
Crypt depth, μm	193	199	177	179	12	0.774	0.110	0.856
VH:CD	1.21	1.21	1.28	1.17	0.05	0.226	0.807	0.268
Viscosity, mPa · s	2.20	2.02	2.13	2.36	0.27	0.617	0.917	0.453

^a^ Antibiotics, phase 1:0.20% of Pennchlor 100, 0.18% of Denagard, 0.25% of zinc oxide; and phase 2 and 3:0.13% of Mecadox 10; 0.25% of zinc oxide.

^b^ β‐glucan, 0.02% of Algamune (Algal Scientific Corp., Plymouth, MI).

The use of antibiotics tended to increase (*p* = 0.066) TNF‐α in the jejunum. The use of antibiotics decreased (*p* < 0.05) IgG in the duodenum and tended to decrease (*p* = 0.063) IgG in the ileum. Supplementation of β‐glucan tended to increase IgA (*p* = 0.078) and TNF‐α (*p* = 0.077) in the plasma (Table 4). Supplementation of β‐glucan reduced (*p* < 0.05) IgA in the jejunum and tended to reduce (*p* = 0.053) IgA in the ileum. No other effects of antibiotics or β‐glucan supplementation on the immune response or oxidative damage products were observed.

**TABLE 4:** Immune responses and oxidative damage products of nursery pigs fed diets supplemented with antibiotics and β‐glucan from microalgae.

Antibiotics^a^	No		Yes		SEM	*p*		
β‐glucan^b^	No	Yes	No	Yes		Antibiotic	β‐glucan	Interaction
Duodenum, unit/mg protein								
Protein carbonyl, nmol	3.09	3.79	3.31	3.77	0.39	0.800	0.141	0.764
IgA, μg	0.014	0.014	0.015	0.016	0.003	0.627	0.984	0.950
IgG, μg	0.015	0.013	0.010	0.009	0.002	0.046	0.478	0.953
TNF‐α, pg	4.15	4.48	5.74	4.39	0.84	0.398	0.567	0.347
Jejunum, unit/mg protein								
Protein carbonyl, nmol	3.82	3.39	3.55	3.89	0.50	0.800	0.924	0.394
IgA, μg	0.006	0.003	0.005	0.003	0.001	0.309	0.021	0.644
IgG, μg	0.013	0.009	0.013	0.010	0.005	0.938	0.334	0.924
TNF‐α, pg	2.31	2.34	2.88	2.95	0.81	0.066	0.254	0.275
Ileum, unit/mg protein								
Protein carbonyl, nmol	4.57	4.49	4.57	3.85	0.46	0.420	0.321	0.412
IgA, μg	0.042	0.034	0.042	0.030	0.005	0.706	0.053	0.660
IgG, μg	0.020	0.018	0.014	0.010	0.003	0.063	0.372	0.780
TNF‐α, pg	13.61	9.45	7.67	10.39	2.81	0.799	0.380	0.227
Plasma, unit/mL								
IgA, mg	0.34	0.43	0.34	0.38	0.04	0.540	0.078	0.483
IgG, mg	6.15	5.75	6.24	5.63	0.71	0.972	0.397	0.857
TNF‐α, pg	234.6	279.7	207.4	238.2	23.0	0.110	0.077	0.733

^a^ Antibiotics, phase 1: 0.20% of Pennchlor 100, 0.18% of Denagard, 0.25% of zinc oxide; and phase 2 and 3: 0.13% of Mecadox 10; 0.25% of zinc oxide.

^b^ β‐glucan, 0.02% of Algamune (Algal Scientific Corp., Plymouth, MI).

## 4 Discussion

The use of antibiotics as growth promoters is no longer widely practiced within the food animal industry, as this practice was associated with the development of antimicrobial resistance (Allen et al. 2013). A potential growth promoter as an alternative to the use of antibiotics may be β‐glucans, which have been shown to improve growth performance (Dritz et al. 1995; Hahn et al. 2006), illicit an intestinal immune response (Baert et al. 2015; Sonck et al. 2010), and influence intestinal morphology and epithelial integrity (Wu et al. 2021) in swine. The aforementioned performance studies were conducted utilizing the β‐glucan derived from yeast, which have a different β‐glucan composition compared with microalgae (Kim 2021; Choi and Kim 2023), as currently, studies are lacking regarding the use of microalgae‐derived β‐glucan in animal feed, specifically.

In the current study, dietary supplementation of β‐glucan derived from microalgae improved the ADG of pigs not fed antibiotics during the first 5 weeks after weaning by 14% compared with pigs not receiving supplemental β‐glucan. Similarly, in the pigs not receiving antibiotics, dietary supplementation of β‐glucan resulted in some improvements to ADFI and G:F in the later weeks of the study. These improvements in growth performance may be due to positive effects of β‐glucan on the intestinal immune system and overall intestinal health. In a study by Hahn et al. (2006), it was found that yeast‐derived β‐glucan tended to increase the ADG of nursery pigs fed β‐1,3/1,6‐glucan at 0.04% of the diet; however, there were no effects on ADFI or G:F. Similarly, Wang et al. (2008) showed an increase in ADG and G:F when nursery pigs received a yeast‐derived β‐1,3/1,6‐glucan at 0.005% of the diet. Other studies have seen similar improvements in growth performance when using yeast‐derived β‐1,3/1,6‐glucan in the diets of weaned pigs (Dritz et al. 1995; Li et al. 2006; Zhou et al. 2013). It is important to note that the cell walls of *Euglena gracilis* differ in β‐glucan structure when compared with β‐glucan derived from yeast. *Euglena gracilis* primarily contain β‐glucan in the form of linear glycosidic links of β‐1,3‐glucan in the main chain, with minor amounts of branching and β‐1,6 glycosidic bonds (Barsanti et al. 2001; Barsanti and Gualtieri 2020). In contrast, β‐glucans from yeast consist of linear β‐1,3‐glucan glycosidic bonds with long β‐1,6‐glucan branches, although the degree of branching differs depending on the yeast source (Caseiro et al. 2022; Manabe and Yamaguchi 2021). It is known that β‐glucan derived from different sources can have different mechanisms of action in the intestine of pigs (Sweeney et al. 2012); however, the lacking body of research surrounding these specific mechanisms indicates that this is an area warranting further investigation.

Intestinal morphology can be influenced by a variety of factors including age, diet, communities within the microbiota, and health status, and morphology can be related to the efficiency of nutrient absorption and growth performance (Cera et al. 1988; Pluske, Hampson, and Williams 1997). The use of select feed ingredients can promote the growth of potentially beneficial microorganisms in the intestines, which may interact with the intestinal immune system and promote intestinal health (Duarte and Kim 2022). For example, studies using algal β‐glucans have shown benefits both in enhancing the integrity of the intestinal barrier (S. W. Kim, Holanda, et al. 2019) and in reducing the populations of potentially harmful species of *Enterobacteria* in the ileum and proximal colon (Sweeney et al. 2012). It was also found that the use of β‐glucans derived from seaweed decreased the populations of potentially harmful *Escherichia coli* , while increasing *Lactobacillus* populations in weaned pigs, which could contribute to decreased intestinal damage and inflammation, and improved nutrient utilization and growth performance (Zou et al. 2021). Similarly, these effects have also been observed with the use of β‐glucans from cereal sources (Reilly et al. 2010; Metzler‐Zebeli et al. 2011); therefore, the general use of β‐glucans could reduce the populations of potentially harmful bacteria in the intestine, enhance recovery of intestinal function following a stress event, and improve intestinal morphology. In the present study, the use of dietary β‐glucans increased villus height in the duodenum of pigs. At the same time, the crypt depth in the duodenum was unaffected by the administration of β‐glucans, possibly due to a slower migration rate of enterocytes along the villi, thereby increasing the villus height and not affecting crypt depth (van Nevel et al. 2003), which improves digestive and absorptive capacities of the small intestine (Pluske, Hampson, and Williams 1997). In agreement with the results of this study, Van Nevel et al. (2003) and Shao, Guo, and Wang (2013) reported that administration of β‐glucans resulted in increased villus height in the jejunum of nursery pigs and broiler chickens, respectively. In contrast, several studies investigating the effects of β‐glucans on intestinal morphology found no increase in villus height in the small intestine when compared with the control (dos Anjos et al. 2019; Wu et al. 2021; Zhou et al. 2022); however, these confounding results could be due to both the variety in sources of β‐glucans and the possible additive effects of additional bioactive materials that may be contained within the β‐glucan sources.

Dietary β‐glucans are widely known for their immunomodulatory effects, primarily due to their interactions with immune cells, like macrophages. The β‐glucans are able to bind to specific surface carbohydrate receptors, such as CR‐3 and Dectin‐1, expressed on the surface of several types of antigen‐presenting cells. (Vetvicka, Vannucci, and Sima 2014). This act of binding to the antigen‐presenting cells can trigger the production of pro‐inflammatory cytokines, such as TNF‐α (Sonck et al. 2010; Wang et al. 2008), and can contribute to overall disease resistance in pigs (Chuaychu et al. 2024). A previous study using weaned pigs challenged with enterotoxigenic *E. coli* , demonstrated that 0.05% of β‐glucan derived from yeast reduced IgA and IgG in serum, and IgA in jejunum, which could suggest that the exposure of β‐glucan to antigen‐presenting cells could equip the immune system to control infections more efficiently (Stuyven et al. 2009). Interestingly, in a study involving healthy humans, Lehne et al. (2005) demonstrated that plasma levels of IgA and IgG were not influenced by dietary β‐1,3‐glucan at 100 mg/day, although 400 mg/day did increase IgA in saliva. In the present study, β‐glucan reduced IgA in small intestine, while tending to increase IgA in plasma. The inconsistent response of the humoral immune system to β‐glucan could be confounded by variables such as source of β‐glucan, and the effects may be less clear in an unchallenged model; therefore, additional studies should be conducted to better understand the influence of β‐glucan from microalgae.

In the present study, β‐glucan supplementation did not affect TNF‐α concentration in the intestinal mucosa; however, it tended to increase TNF‐α concentration in plasma. This result agrees with an in vitro study by Sonck et al. (2010), in which an increase in TNF‐α secretion was observed when β‐1,3‐glucan and β‐1,3/1,6‐glucan were provided to peripheral blood mononuclear cells and neutrophils of pigs. Similarly, Adams et al. (2008) suggest that the presence of structures other than β‐1,3‐glucan residues in the backbone could limit interactions with Dectin‐1 molecules, resulting in lower affinity. This indicates that β‐1,3‐glucan alone, like the structures found in *E. gracilis*, might be a more effective in stimulating the immune system, compared with other sources that contain β‐1,3/1,6‐glucan.

The results of this study highlight the potential effects of microalgae‐derived β‐glucans as a promising alternative to antibiotics due to their ability to improve growth performance and intestinal health in pigs. Dietary supplementation with β‐glucans demonstrated improvements in growth performance metrics, potentially due to improvements in intestinal morphology. The effects could be attributed to the interactions of β‐glucans with the antigen‐presenting cells found in the intestine, bolstering the immune system and helping pigs adapt to challenging environments. However, inconsistencies in the immune responses indicate that further research is needed to fully understand the mechanisms by which β‐glucans can positively influence the intestinal immune system.

## Conflicts of Interest

The authors declare no conflicts of interest.
